# Complex Formation of Resorufin and Resazurin with Β-Cyclodextrins: Can Cyclodextrins Interfere with a Resazurin Cell Viability Assay?

**DOI:** 10.3390/molecules23020382

**Published:** 2018-02-10

**Authors:** Rita Csepregi, Beáta Lemli, Sándor Kunsági-Máté, Lajos Szente, Tamás Kőszegi, Balázs Németi, Miklós Poór

**Affiliations:** 1 Department of Laboratory Medicine, University of Pécs, Medical School, Pécs H-7624, Hungary; ritacsepregi93@gmail.com (R.C.); koszegi.tamas@pte.hu (T.K.); 2 János Szentágothai Research Center, University of Pécs, Pécs H-7624, Hungary; lemli.beata@gytk.pte.hu (B.L.); kunsagi-mate.sandor@gytk.pte.hu (S.K.-M.); 3 Department of General and Physical Chemistry, University of Pécs, Pécs H-7624, Hungary; 4 Department of Pharmaceutical Chemistry, University of Pécs, Faculty of Pharmacy, Pécs H-7624, Hungary; 5 CycloLab Cyclodextrin Research & Development Laboratory, Ltd., Budapest H-1097, Hungary; szente@cyclolab.hu; 6 Department of Pharmacology, University of Pécs, Faculty of Pharmacy, Pécs H-7624, Hungary; balazs.nemeti@aok.pte.hu

**Keywords:** resazurin, resorufin, cyclodextrin, host-guest interaction, Alamar Blue, cell viability assay

## Abstract

Resazurin (or Alamar Blue) is a poorly fluorescent dye. During the cellular reduction of resazurin, its highly fluorescent product resorufin is formed. Resazurin assay is a commonly applied method to investigate viability of bacterial and mammalian cells. In this study, the interaction of resazurin and resorufin with β-cyclodextrins was investigated employing spectroscopic and molecular modeling studies. Furthermore, the influence of β-cyclodextrins on resazurin-based cell viability assay was also tested. Both resazurin and resorufin form stable complexes with the examined β-cyclodextrins (2.0–3.1 × 10^3^ and 1.3–1.8 × 10^3^ L/mol were determined as binding constants, respectively). Cells were incubated for 30 and 120 min and treated with resazurin and/or β-cyclodextrins. Our results suggest that cyclodextrins are able to interfere with the resazurin-based cell viability assay that presumably results from the following mechanisms: (1) inhibition of the cellular uptake of resazurin and (2) enhancement of the fluorescence signal of the formed resorufin.

## 1. Introduction

**Figure 1:**
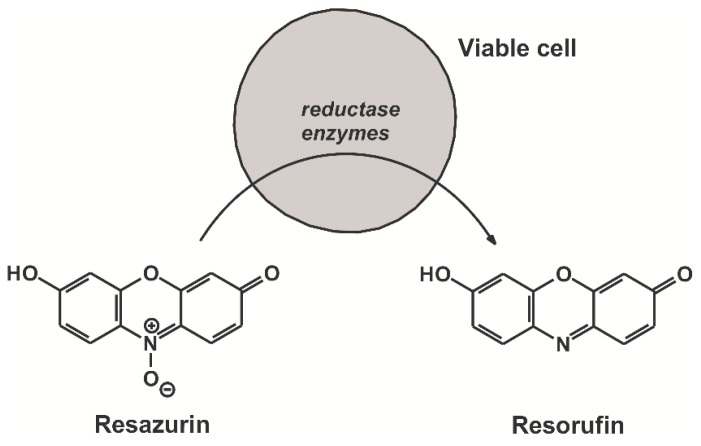
Chemical structures of resazurin and resorufin. Reductases of viable cells reduce resazurin resulting in the formation of its highly fluorescent metabolic product resorufin.

Resazurin (7-hydroxy-3H-phenoxazin-3-one 10-oxide) also called Alamar Blue is a dye commonly used for measurements assaying cell viability [1,2,3]. Resazurin itself is weakly fluorescent; however, its reduction by bacteria or mammalian cells results in a pink and highly fluorescent derivative resorufin (7-hydroxy-3*H*-phenoxazin-3-one) that is formed (Figure 1) [4]. Resorufin in turn can be reduced further into the colorless and non-fluorescent hydroresorufin [5]. The resazurin assay is applied commonly to investigate antibiotic resistance of some bacteria (e.g., *Mycobacterium* strains), to test the antibacterial action of various compounds, or even to examine bacterial biofilm formation [6,7,8]. On comparing with other assays testing cell viability, the resazurin-based assay appears suitable and reliable for investigating the toxic effect of various compounds on mammalian cells [2,9,10]. Based on previous studies, there is a direct correlation between the reduction of resazurin and the number/proliferation of bacteria or mammalian cells [5]. The resazurin or Alamar Blue assay is used commonly for testing cell viability because its cost is low, and we do not need to extract the cells, owing to the much lower cytotoxicity of resazurin and its derivatives than of the reagents employed in other assays, including the commonly applied MTT assay [5]. Resazurin assay itself is a simple and rapid method to test cell viability. Resazurin solution is added to the cells in a volume corresponding to 10% of the medium (without replacing the medium), and then the conversion to resorufin can be measured by either colorimetry or fluorimetry. Nevertheless, fluorimetry appears more sensitive compared to the colorimetric analysis [5]. The fluorescence excitation and emission maxima of resorufin are approximately at 570 nm and 585 nm, respectively [11]. However, during the resazurin-based viability assay, the fluorescence of the formed resorufin is determined in the samples using 530 to 580 nm as excitation and 570 to 620 nm as emission wavelengths [3,10,12].

Cyclodextrins (CDs) are intensively studied host molecules that are widely applied by analytical chemistry as well as food, cosmetic, and pharmaceutical industries [13,14,15,16]. The usually employed CDs are α-, β-, and γ-cyclodextrins, which are built up from six, seven, or eight glucopyranose units, respectively [17,18]. CDs possess a ring-shaped, conical structure with a hydrophobic interior and a hydrophilic exterior spaces, which makes their internal cavity able to accommodate relatively lipophilic molecules or structural moieties. The stability of the formed host-guest type complexes as well as the selectivity of CDs towards the guest molecules are usually highly influenced by chemical modifications of the basic CD structures [18,19].

Based on previous investigations, resazurin forms a stable complex with γ-CD [20], and resorufin interacts with native β- and γ-cyclodextrins [21,22,23]. CDs are able to influence the cellular uptake of the guest molecules [24]; furthermore, CD-complexes of fluorophore molecules commonly exhibit stronger fluorescence than the fluorophore alone [25,26]. A recent study reported that the presence of CDs can interfere with bioluminescence imaging due to complex formation with D-luciferin [27]. Therefore, CDs may also be able to disturb the measurement of other fluorescent dye molecules, including resorufin. CDs can occur in several in vitro experiments carried out on cells where the resazurin-based assay is applied to test cell viability [28,29,30]. The basic protocol of the resazurin assay applies addition of 10% volume of resazurin solution to the cell medium without the replacement of the medium. This, however, may result in the interaction of CDs with resazurin and/or the formed resorufin. Even if many researchers replace the cell media (and thus the CDs present) before resazurin assay, methylated CDs can be taken up by cells through fluid-phase endocytosis [31], resulting in the possible formation of resazurin-CD and resorufin-CD complexes intracellularly.

In this study, the complex formation of resorufin and the parent compound resazurin with β-cyclodextrin (BCD), hydroxypropyl-β-cyclodextrin (HPBCD), and heptakis-2,6-di-*O*-methyl-β-cyclodextrin (DIMEB) was investigated employing fluorescence and UV-Vis spectroscopy. The fluorescence enhancement of resorufin by CDs as well as the stability of the formed resorufin-CD complexes were determined, and the interaction was investigated further by molecular modeling studies. Then resazurin-CD and resorufin-CD complex formations were examined employing UV-Vis spectroscopy. Because our spectroscopic studies suggested the strong fluorescence enhancement of resorufin by CDs as well as the formation of stable dye-CD complexes, the effect of CDs on resazurin-based cell viability assay was also tested. As the results demonstrate, some of the CDs are able to interfere with resazurin assay. Therefore, it should also be taken into account that when assaying cell viability, CDs present in the medium could potentially confound the data obtained.

## 2. Results and Discussion

### 2.1. Fluorescence Spectroscopic Investigation of Resorufin-CD Interactions

**Figure 2:**
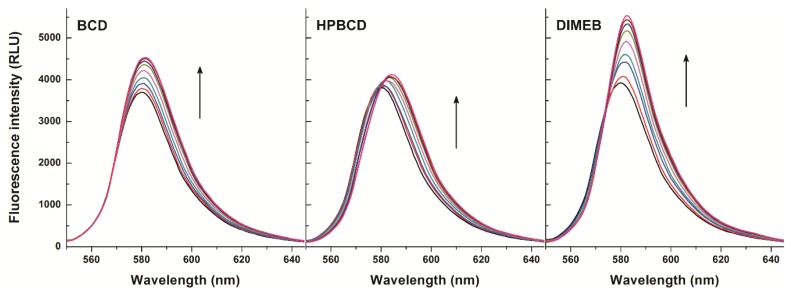
Fluorescence emission spectrum of resorufin (0.4 μM) in the presence of cyclodextrins (CDs) added at concentrations 0, 50, 100, 200, 400, 600, 800, 1000, and 1200 μM in PBS (pH 7.4; λ_ex_ = 570 nm) (RLU = relative light unit).

First, the concentration dependent effect of CDs on the fluorescence signal of resorufin was examined at 25 °C. Therefore, increasing concentrations of BCD, HPBCD, and DIMEB (0–1200 μM) were added to a fixed concentration of resorufin (0.4 μM) in phosphate-buffered saline (PBS, pH 7.4). Fluorescence emission spectra were recorded using 570 nm as excitation wavelength (this wavelength proved the excitation maximum of resorufin; see in Figure S1). As Figure 2 demonstrates, the fluorescence signal of resorufin increased in the presence of each CD in a concentration dependent fashion. The strongest fluorescence enhancement of resorufin was observed with DIMEB, while in the presence of BCD and HPBCD, the fluorescence enhancement was weaker. Furthermore, a red shift of the emission maximum of resorufin was observed in the presence of each of the three CDs (HPBCD > DIMEB > BCD). Under the applied conditions, CDs alone did not express any fluorescence signal; therefore, it is reasonable to hypothesize that the fluorescence enhancement of resorufin by CDs is ascribable to the host-guest type complex formation of the examined compounds. The microenvironment within the cyclodextrin cavity is less polar than in water; its dielectric properties are close to those of 70% *v*/*v* ethanol-water mixture. The fluorescence signal of a fluorophore is strongly influenced by polarity of the environment around the molecule. In the CD nanocavity, the fluorophore is surrounded by apolar microenvironment, thus affecting its fluorescence due to the molecule being entrapped by the CD. Based on these principles, complex formation of a fluorophore with a CD is commonly associated with the increase of its fluorescence signal [25,26]. Furthermore, the methylation of BCD has two consequences: (1) the cavity depth is extended in both primary and secondary side of the cyclinder, which becomes taller, while cavity diameter is unchanged and (2) self-assembly-related poor aqueous solubility of parent BCD is dramatically improved by methylation (intermolecular H-bond formation is disrupted by methyl groups).

Enhancement of the fluorescence emission intensity of resorufin was evaluated at three emission wavelengths (580, 585, and 590 nm). As Table 1 demonstrates, in the presence of 0.4 μM resorufin and 1200 μM of CDs 22–38%, 1–39%, and 37–53% increases of the fluorescence signal of resorufin were noticed as a result of the complex formations with BCD, HPBCD, and DIMEB, respectively. These observations suggest that during resazurin-based cell viability assays CDs can interact with the formed resorufin. This complex formation, however, leads to a significant increase of the fluorescence signal. Considering the fluorescence enhancement of resorufin by CDs, the resorufin-CD interaction can most likely confound the results of the resazurin-based cell viability assay. This phenomenon may be the most prominent with DIMEB because, being a methylated CD molecule, cells take it up by fluid-phase endocytosis [31]. Moreover, as demonstrated in Table 1, DIMEB induced the strongest fluorescence enhancement of resorufin at each tested emission wavelengths, complicating the evaluation of data even further. With respect to the other CDs tested, they have not been reported to cross the cell membrane, likely limiting their presence to the cell medium.

**Figure 3:**
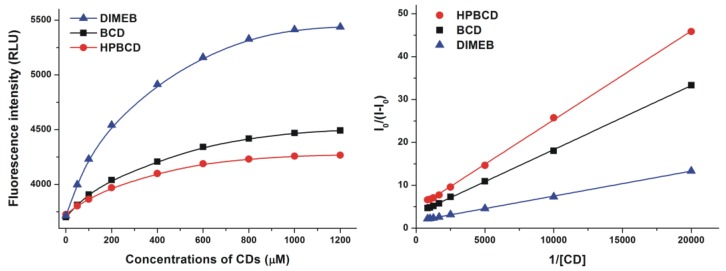
Fluorescence emission intensities (left) of resorufin (0.4 μM) in the presence of increasing β-cyclodextrin (BCD), hydroxypropyl-β-cyclodextrin (HPBCD), and heptakis-2,6-di-*O*-methyl-β-cyclodextrin (DIMEB) concentrations in PBS (pH 7.4; left). Benesi-Hildebrand plots of resorufin-CD complexes (right; λ_ex_ = 570 nm, λ_em_ = 583 nm) (RLU = relative light unit).

The fluorescence of the resorufin-CD complexes was significantly stronger than that of the dye molecule in its free form. Based on the enhanced fluorescence of resorufin in the presence of increasing CD concentrations (Figure 3, left), the binding constants of the formed complexes were calculated employing the graphical application of the Benesi-Hildebrand equation (Equation (1)). Benesi-Hildebrand plots of resorufin-CD complexes were linear and correlated with the 1:1 stoichiometry model well (Figure 3, right). The calculated binding constants of the tested resorufin-CD complexes indicated similar stability values; however, HPBCD and DIMEB formed more stable complexes with resorufin compared to the native BCD molecule (Table 1). Our results are in good agreement with the previously published data of Balabai et al. [21], where similar log*K* value of resorufin-BCD complex (log*K* = 3.3) and 1:1 stoichiometry of complex formation were reported.

Because the incubation of cells with resazurin during the viability assay is carried out at 37 °C, resorufin-CD complex formations were investigated at 37 °C as well. The stability of the complexes was lower at 37 °C than at 25 °C, irrespective of the CD tested. The stability of the resorufin-DIMEB complex weakened minimally, whereas the stability of the resorufin complexes with the other two CDs exhibited a more pronounced, although still slight, reduction (Table 1). These findings indicate that we can count on similar stability of the resorufin-CD complexes at 37 °C as at 25 °C.

### 2.2. Molecular Modeling of Resorufin-CD Interactions

Host-guest interactions are known to be associated with the loss of solvent shell of guest molecules and host’s cavity becoming empty prior to the molecular association. Since the experimental results alone cannot confirm the dehydration of the guest molecules, both situations (hydrated or dehydrated guest) were considered during molecular modeling. Accordingly, three series of calculations were performed: (1) the dehydration was ignored during the determination of the enthalpy and entropy changes of the molecular interactions; (2) the dehydration of the guest molecules was considered and calculated by the TIP3P model; and (3) both the dehydration and the energy cost associated to the exit of water molecules from BCD and DIMEB cavities prior the interaction with the resorufin molecules were considered (Table 2).

Very similar Gibbs free energy values were obtained at 298 K in both the hydrated and the dehydrated guest model, clearly showing that the experiments cannot offer information about the hydration state. However, the molecular dynamics simulation are in accordance with the enthalpy-entropy compensation effect associated to the dehydration of the guest: the energy invested to remove the hydration shell of resorufin molecules appears in the entropy gain associated to the molecular interaction. The negative Gibbs free energy changes validated the presence of stable resorufin-BCD and slightly stronger resorufin-DIMEB complexes in the solutions at room temperature. The formation of stable complexes is supported by both the negative enthalpy and the positive entropy terms, independently from the fact whether or not the hydration is considered during the simulations.

The stability values calculated from Gibbs free energy changes (Table 2) are approximately three order of magnitude higher compared to the experimental values. These findings reflect the presence of exchange reactions instead of simple molecular association. To model plausible processes, the theoretical calculations were extended for the following model: three water molecules located in the cavity of the BCD or DIMEB leave before the resorufin guest enters into the cavity. Calculations show markedly less enthalpy changes for these exchange reactions, while the entropy values do not change significantly, compared to the Δ*H* and Δ*S* values associated to the guest dehydration model (Table 2). The reduction of the enthalpy changes can be explained by the breaking of the bonds between the water molecules and the host’s cavity, while the unchanged entropy values reflect ordered structure of water molecules both inside and outside the host’s cavity. The theoretically calculated stability constants derived from the third model are in good agreement with the experimentally determined data and exhibit the same tendency: the stability of the resorufin-DIMEB complex is higher than that of the resorufin-BCD complex.

**Figure 4:**
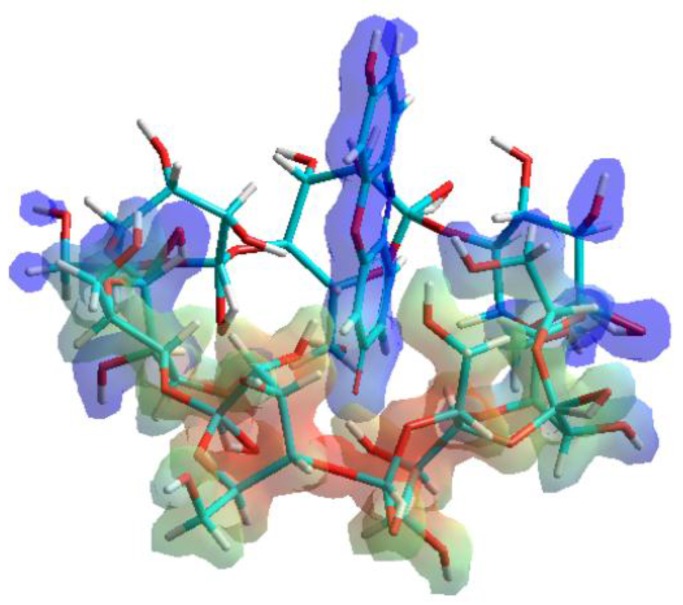
Inclusion complex of the host β-cyclodextrin (BCD) molecule with the resorufin guest. The complex formation is based on the interaction between the benzoquinone-imine moiety of resorufin molecule and the apolar BCD cavity.

In agreement with the well-known enthalpy-entropy compensation effect, the decomposition of the solvation shell of resorufin molecules does not affect the formation of resorufin-BCD or resorufin-DIMEB complexes around room-temperature. The enthalpy-entropy compensation effect is associated to the energy cost of breaking the interaction between the solvent molecules and the solute, during which the solvation shell is dissipated. The entropy gain derived from the increased freedom of solvent molecules becomes released from the solvation shell of the resorufin molecule immediately before the complex formation. On calculation, the protonated and deprotonated forms of resorufin returned the same interaction energy changes (data not shown). This phenomenon is likely due to the inclusion of the benzoquinone-imine moiety, which remains practically unaffected by the presence or absence of the proton of the OH group (Figure 4).

### 2.3. Investigation of Resazurin-CD and Resorufin-CD Interactions with UV-Vis Spectroscopy

**Figure 5:**
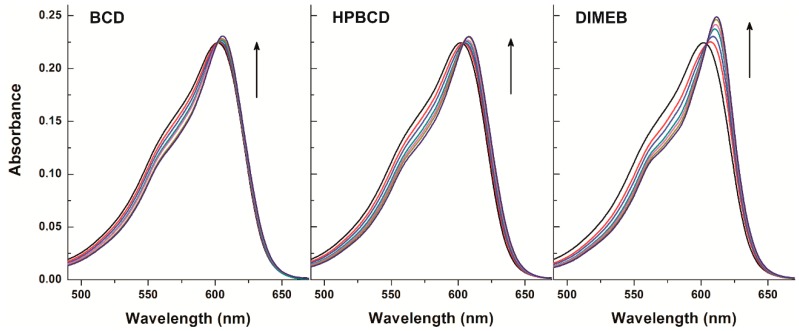
Absorption spectra of resazurin (4 μM) in the presence of cyclodextrins (CDs) added at concentrations 0, 250, 500, 1000, 1500, 2500, and 4000 μM in PBS (pH 7.4).

**Figure 6:**
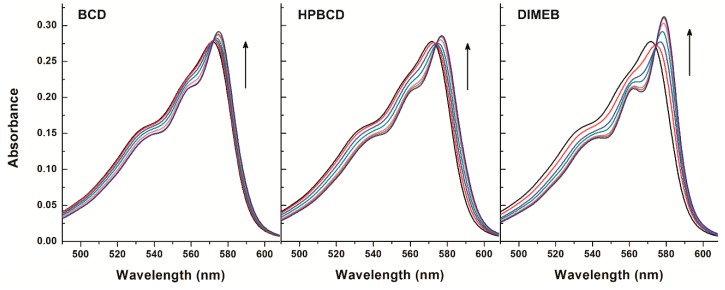
Absorption spectra of resorufin (4 μM) in the presence of cyclodextrins (CDs) added at concentrations 0, 100, 250, 500, 1000, 1500, and 2000 μM in PBS (pH 7.4).

To confirm the calculated binding constants of resorufin-CD complexes (determined from the data of fluorescence spectroscopy; see in Section 2.1) as well as to investigate the possible formation and stability of resazurin-CD complexes, absorption spectra of resorufin and the parent compound resazurin were recorded in the presence of CDs at 25 °C. CDs (0–4000 μM) were added to a fixed concentration of resorufin or resazurin (both 4 μM) in PBS. The absorbance values of both resorufin and resazurin increased together with the increasing CD concentrations (Figure 5 and Figure 6, respectively). Importantly, CDs alone (that is, without resazurin and resorufin) did not have any absorbance at the applied concentrations (data not shown), indicating that the abovementioned increase in the absorbance is most likely due to the interaction of resazurin or resorufin with CDs. In addition, not only absorbance increase but also a slight red shift of the absorption maxima was observed (Figure 5 and Figure 6). These findings suggest that resorufin and resazurin are able to form complexes with CDs.

**Figure 7:**
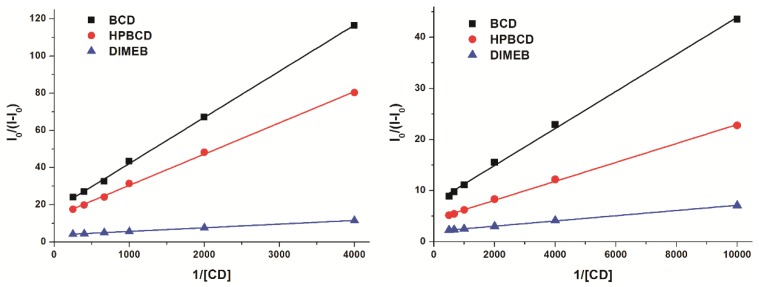
Benesi-Hildebrand plots of resazurin-CD (left; 4 μM resazurin + 0, 250, 500, 1000, 1500, 2500, and 4000 μM concentrations of cyclodextrins (CDs)) and resorufin-CD (right; 4 μM resorufin + 0, 100, 250, 500, 1000, 1500, and 2000 μM concentrations of CDs) complexes (see wavelengths given in Table 3).

To support this further, binding constants of the resorufin-CD and resazurin-CD complexes were calculated, employing the graphical application of the Benesi-Hildebrand equation (Equation (1)). As Figure 7 demonstrates, a linear correlation corresponding to 1:1 stoichiometry of the formed complexes was seen. Binding constants of resorufin-CD complexes (Table 3) were in good agreement with from the findings of the fluorescence spectroscopic studies (Table 1). Furthermore, similar log*K* values were calculated for the resazurin-CD than for the resorufin-CD complexes (Table 3), confirming the conclusion that resazurin can indeed form complexes with CDs with similar stability than resorufin. These observations strongly suggest that CDs present in cell media are able to influence the cellular uptake of the resazurin through complex formation. The velocity and the extent of the cellular uptake of resazurin is a crucial part of the viability assay because intracellular reductases convert the parent compound resazurin to the highly fluorescent product resorufin. Therefore, CDs may influence resazurin-based cell viability assay in two separate ways: (1) the modified fluorescence intensity of the intracellularly formed resorufin because of its complex formation with CDs (applies only for DIMEB of the tested CDs that is taken up by cells) and (2) the extracellular complex formation of the parent compound resazurin with CDs may also modify the cellular uptake of resazurin.

### 2.4. Effects of CDs on Resazurin-Based Cell Viability Assay, and on ATP and Total Protein Levels of HepG2 Cells

**Figure 8:**
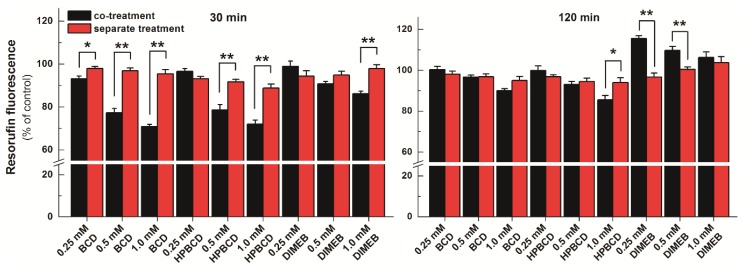
Resazurin-based cell viability assay on HepG2 cells. The cells were incubated with cyclodextrins (CDs) together with resazurin (black bars) or with CDs followed by resazurin (red bars) for 30 min (left) or 120 min (right). Bars represent mean ± SEM of five independent experiments, based on the measurement of resorufin (* *p* < 0.05, ** *p* < 0.01).

To test the effect of CDs on resazurin-based cell viability assay, cells were treated for 30 or 120 min with 0, 0.25, 0.5, and 1.0 mM CD concentrations in the presence of 2 μM resazurin (co-treatment). Furthermore, because CDs may be able to influence the viability of HepG2 cells [25], it was also investigated how treatment of cells separately (first with CDs then, following the removal of CDs from the medium, with resazurin) affects cell viability. Figure 8 demonstrates the results of co-treatments and separate incubations after 30 or 120 min. In the 30-min incubations, 0.5 and 1.0 mM BCD and HPBCD concentrations led to significantly lower resorufin signals in the co-treated cells than in cells treated separately (where the CD-containing medium was replaced with CD-free medium before the resazurin assay). Similar effect was observed in the presence of 1.0 mM DIMEB, albeit somewhat weaker (Figure 8, left). Based on our current knowledge, CDs are not able to directly influence the cellular action of a compound; however, the entrapment of a guest molecule inside the CD cavity can inhibit its binding to a target structure (e.g., proteins) or can influence the transport of the guest molecule through the cell membrane. Purportedly, CDs reduce the cellular uptake of resazurin and in turn its intracellular conversion to the highly fluorescent derivative resorufin, because CDs are able to form stable complexes with the parent compound resazurin extracellularly (see in Figure 7 and Table 3), preventing its entry into the cell. This idea is supported by the fact that the cellular uptake (including the gastrointestinal absorption) of drugs whose CD complexes are with relatively low binding constants (log*K* is typically 2 to 3) is enhanced by CDs [16], while formation of such host-guest complexes with higher binding constants can result in poor cellular uptake of the guest [25]. Thus, the calculated log*K* values of resazurin-CD complexes (Table 3) may explain the observed effects. DIMEB brought about smaller differences between the co-treated and the separately treated cells in the 30-min incubations. This might originate from the cellular uptake of DIMEB, because its intracellular presence can, at least partly, alleviate the observed CD effects due to enhancing the fluorescence signal of the intracellularly formed resorufin.

The difference observed in 30-min incubations between co-treated and separately treated cells almost completely disappeared in incubations lasting for 120 min, as only minor differences were observed in the presence of 1.0 mM BCD and 1.0 mM HPBCD concentrations (Figure 8, right). In contrast, DIMEB, instead of weakening the fluorescence seen after 30 min incubation, increased the signal intensity significantly in cells co-treated with resazurin and DIMEB compared to cells treated separately (Figure 8). This phenomenon is likely due to the enhancement of the fluorescence of the intracellularly formed resorufin by DIMEB, because DIMEB belongs to the methylated CDs, which are known to be taken up by cells through endocytosis, whereas other CDs cannot cross the cell membrane [31]. The longer incubation period can readily result in higher amounts of resorufin formed within and higher amount of DIMEB taken up by the cells, leading to increased intracellular resorufin-DIMEB complex formation and stronger fluorescence enhancement. Interestingly, the difference between co-treated and separately treated cells decreased in response to increasing DIMEB concentrations. This phenomenon may also come from the increased cellular uptake of DIMEB during the 120 min incubation period. Even if the medium was replaced before resazurin assay, increasing intracellular DIMEB concentrations can result in three consequences: (1) viability loss of HepG2 cells; (2) increased complex formation with resazurin, thus preventing the reduction of the parent compound; and (3) increased fluorescence signal of resorufin due to the complex formation. Since the loss of cell viability and the intracellular resazurin-DIMEB complex formation counter the increased fluorescence signal of resorufin, this can explain why increased DIMEB concentrations led to weaker enhancement of the resorufin signal.

**Figure 9:**
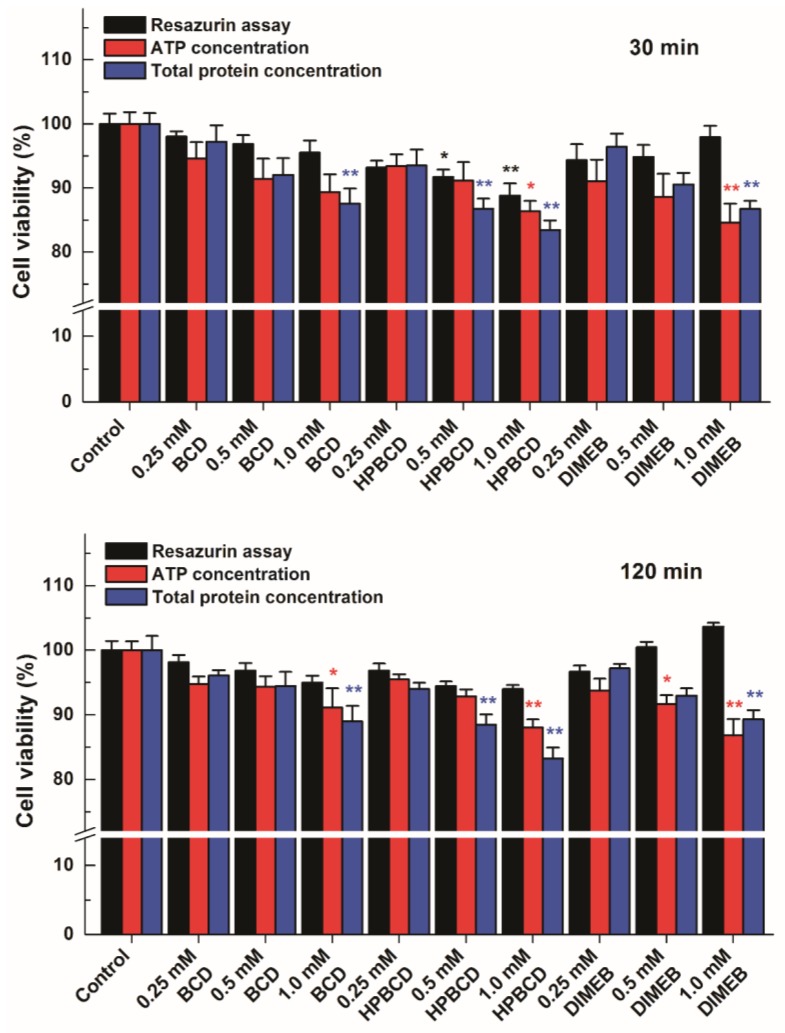
Comparison of the results of the resazurin-based cell viability assay in HepG2 cells treated first with cyclodextrins (CDs) then with resazurin (that is, the CD-containing medium was replaced before addition of resazurin) with the data originating from the changes of intracellular ATP and total protein levels. The cells were incubated for 30 (**top**) or 120 min (**bottom**) with CDs (β-cyclodextrin (BCD), hydroxypropyl-β-cyclodextrin (HPBCD), or heptakis-2,6-di-*O*-methyl-β-cyclodextrin (DIMEB)). Bars represent mean ± SEM of five independent experiments. Asterisks indicate significant difference compared to control incubations with no CD added (* *p* < 0.05, ** *p* < 0.01).

In order to test this hypothesis, HepG2 cells were incubated for 30 or 120 min with CDs, and then the medium was removed. The thus obtained cell cultures were used for measuring ATP and total protein levels of cells and the results were compared with the data of the resazurin-based assay. Both ATP and total protein levels showed that CD treatments led to a slight loss of viability of HepG2 cells, correlating well with the resazurin assay in cells treated with BCD or HPBCD (Figure 9). In addition, both ATP and total protein levels suggest the loss of cell viability in the presence of DIMEB, even though this CD caused a concentration-dependent increase of the resorufin signal. These results support the idea that despite DIMEB-containing cell medium was removed from HepG2 cells, during the incubations some DIMEB molecules were taken up by cells. Therefore, the intracellular presence of DIMEB may result in the formation of resorufin-DIMEB complexes that can lead to increased fluorescence signal (even though the cell viability suffers some loss), and consequently yields false data in the resazurin-based cell viability assay.

CDs can enhance the aqueous solubility of lipophilic molecules and/or enhance their cellular uptake; therefore, the biological action of CD complexes with different compounds or the cellular uptake of encapsulated guest molecules are commonly tested in cell experiments [32,33,34]. Because the stability of these CD complexes are typically low, micromolar concentrations of guest molecules should be accompanied by millimolar CD concentrations for achieving encapsulation of the guest at considerable level. Furthermore, CDs (mainly methylated derivatives) are commonly used for manipulate cellular cholesterol content or for extraction of cholesterol from cell membranes [35,36,37]. During the above-listed experiments, the effects of the encapsulated test compound and of the applied CD on cell viability need to be tested as well. Since resazurin is a widely used cell viability assay dye, there is a rational chance that researchers choose resazurin to evaluate cell viability.

## 3. Materials and Methods 

### 3.1. Reagents

All reagents were of spectroscopic or analytical grade. Resazurin and resorufin were purchased from Sigma-Aldrich (Waltham, MA, USA). Cyclodextrins, including β-cyclodextrin (BCD), hydroxypropyl-β-cyclodextrin (HPBCD; DS (degree of substitution) = 4.5), and heptakis-2,6-di-*O*-methyl-β-cyclodextrin (DIMEB; DS = 14), were obtained from CycloLab Cyclodextrin Research & Development Laboratory (Budapest, Hungary). 1000 μM resazurin and 1000 μM resorufin stock solutions were prepared in dimethyl sulfoxide (Fluka Analytical, spectroscopic grade, Steinheim, Germany) and stored at −20 °C, protected from light. Dulbecco’s Modified Eagle Medium (DMEM, Sigma-Aldrich, Waltham, MA, USA), Fetal Bovine Serum (FBS, Pan-Biotech, Aidenbach, Germany), Bioluminescent ATP Assay Kit CLSII (Roche, Paris, France), bovine serum albumin (BSA; Biosera, Nuaille, France), and Coomassie Brilliant Blue G-250 (Reanal, Budapest, Hungary) were used as received.

### 3.2. Fluorescence Spectroscopic Measurements

Fluorescence measurements were performed employing a Hitachi F-4500 fluorescence spectrophotometer (Tokyo, Japan). Measurements were carried out at 25 or 37 °C in phosphate buffered saline (PBS, pH 7.4). During these studies, increasing CD concentrations were added to standard amount of resorufin (final concentrations: resorufin = 0.4 μM; CDs = 0, 50, 100, 200, 400, 600, 800, 1000, and 1200 μM), and then fluorescence emission spectra were recorded using 570 nm as excitation wavelength. Binding constants of CD complexes were calculated, using the graphical application of Benesi-Hildebrand equation, assuming 1:1 stoichiometry:

(1) $$\frac{I_{0}}{\left(I-I_{0}\right)}=\frac{1}{A}+\frac{1}{A*K*{\left[CD\right]}^{n}}$$

where *K* is the binding constant (with the unit of L/mol), *I_0_* and *I* are fluorescence emission intensities of dye molecules at 583 nm in the absence and presence of CDs, respectively. *[CD]* is the molar concentration of CDs in the samples, while *A* is a constant and *n* is the number of binding sites.

### 3.3. UV-Vis Spectroscopic Measurements

UV-Vis spectra were recorded using Specord Plus 210 (Analytic Jena AG, Jena, Germany) spectrophotometer. Measurements were carried out at 25 °C in PBS (pH 7.4). During these studies, CDs at concentrations of 0, 250, 500, 1000, 1500, 2500, and 4000 μM were added to standard resazurin at a concentration of 4 μM, and then absorption spectra were recorded. The absorption spectra of resorufin (4 μM) were also measured in the presence of CDs at concentrations of 0, 100, 250, 500, 1000, 1500, and 2000 μM. Since the complex formation of CDs with resazurin and resorufin results in a slight increase of the absorbance of these dye molecules, binding constants of CD complexes were calculated, employing the graphical application of the Benesi-Hildebrand equation (Equation (1); see in Section 3.2), where the fluorescence intensity values were replaced with the absorbance values.

### 3.4. Modeling Studies

Semi-empirical AM1 method was applied to determine the initial structures for molecular dynamics simulations. Regarding DIMEB, the structures used for calculations were generated by replacing hydrogens of two OH groups linked to carbon atoms 2 and 6 of each glucose moieties. Atomic charges of the resorufin molecule and the CDs were calculated using the B3LYP/6-31G(d) method and the basis was set by performing natural population analysis (NPA). Transition states along the reaction path were determined by the HyperChem package (Hypercube, Inc., Gainesville, FL, USA) and the existence of saddle point was validated by the appearance of one virtual vibration frequency. Molecular dynamics calculations were performed by modeling the interactions at room-temperature. Both the liquid and gas-phase simulations were performed using the MM+ force field implemented in the HyperChem 8.0 program package. The liquid environment was considered by the TIP3P model. In order to find an appropriate initial condition for the dynamics calculations, a “heating” algorithm implemented in HyperChem was used. This procedure heats the molecular system smoothly from lower temperatures to the temperature *T*, at which the molecular dynamics simulation is performed. The starting geometry for this heating phase is a static initial structure. We used the optimized AM1 geometry of the molecules interacted as initial structures, and the temperature step and the time step in the heating phase were set to 2 K and 0.1 fs, respectively.

Considering the dominance of molecular vibrations in the entropy term associated to the molecular motions, the change of molecular vibrations during the interaction of resorufin and CD molecules was considered to determine the entropy change associated to the complex formation between the resorufin and CD molecules [38,39]. The overall effect of vibrations on the entropy changes and the vibrational entropy contents of each species were calculated applying the Boltzmann-statistics. Accordingly, the frequencies were calculated in the harmonic approximation, and the entropy was calculated on the common way using HyperChem code (Equation (2)):

(2) $$S_{vib}=R\sum_{i}\{\frac{h\nu_{i}/kT}{e^{\left(h\nu_{i}/kT\right)}-1}-\ln[1-e^{\left(-h\nu_{i}/kT\right)}]\}$$

where *ν_i_* is the frequency of vibration, *T* is the temperature (here equals to 298.16 K). Considering the known limitation of the procedure above [40], the results are applicable within the temperature range of experiments.

The solvation entropy and enthalpy of resorufin were calculated at semi-empirical AM1 level using TIP3P model implemented in the HyperChem code: entropy content calculated for the gas phase molecule was subtracted from the entropy term of solvated species calculated by the TIP3P model. Similar method was applied for the enthalpy term.

### 3.5. Cell Culture

Adherent cell culture (HepG2, human liver hepatocellular carcinoma, ATCC: HB-8065, Teddington, UK) was cultured in DMEM supplemented with 10% FBS, penicillin (100 U/mL) and streptomycin (100 µg/mL), and incubated at 37 °C in a humidified atmosphere containing 5% CO_2_. Cells were then trypsinized and plated into 96-well sterile plastic plates (10^4^ cells/well). After reaching 80% confluency, the culture medium was replaced with fresh medium containing one of the appropriate concentrations (0, 0.25 mM, 0.5 mM, and 1.0 mM) of the selected CDs (BCD, HPBCD, or DIMEB). Both pre-treatment of cells with CDs (before the addition of resazurin) and the co-treatment with resazurin and CDs were performed.

### 3.6. Resazurin-Based Cell Viability Assay

In order to test the influence of CDs on resazurin-based cell viability assay, the medium was replaced with fresh DMEM containing 0, 0.25, 0.5, and 1.0 mM CD concentrations (150 μL medium/well). Immediately after the CD treatment, 15 μL of 22 μM resazurin solution (diluted in DMEM) was added to each well (final concentration of resazurin was 2 μM). Thereafter, cells were incubated for 30 or 120 min at 37 °C in the dark then fluorescence emission of the formed resorufin dye was determined in the samples employing a multimode plate reader (Perkin Elmer EnSpire Multimode reader, Waltham, MA, U.S.) using 560 and 590 nm as excitation and emission wavelengths, respectively.

In order to test the potential viability loss of HepG2 cells resulted from the CD treatment, the experiment described above was repeated with some modifications: (1) Cells were pre-treated with CDs for 30 min then the medium was removed. Thereafter, cells were washed three times with 200 μL PBS then 150 μL fresh DMEM and 15 μL of 22 μM resazurin solution (diluted in DMEM) were added to each well. After 30 min incubation at 37 °C in the dark, resorufin contents were determined by plate reader. (2) Cells were pre-treated with CDs for 120 min then the medium was removed. Thereafter, cells were washed three times with 200 μL PBS then 150 μL fresh DMEM and 15 μL of 22 μM resazurin solution (diluted in DMEM) were added to each well. After 120 min incubation at 37 °C in the dark, resorufin contents were determined by plate reader.

### 3.7. Quantitation of Intracellular ATP and Total Protein Levels

To further demonstrate the effects of CDs on the viability of HepG2 cells following 30 or 120 min incubation with CDs (0, 0.25, 0.5, and 1.0 mM), intracellular ATP and total protein levels were quantified employing our previously published method with minor modifications [41]. After CD treatment, cells were washed three times with PBS then 250 μL of 5% perchloric acid (PCA) was added to each well. After 15 min incubation at room temperature, 150 μL of the PCA extracts were transferred into an empty plate and neutralized with 9.13% potassium hydroxide (100 μL/well), during which samples were kept in ice. After sedimentation of the formed potassium perchlorate precipitate, 10 µL of the supernates were pipetted into 100 µL ATP reagent, using a white 96-well optical plate. The luminescent ATP measurement was carried out using a plate reader and applying 0.2 second as measuring time.

Intracellular total protein levels were determined using the Bradford reagent [41]. Relative protein concentrations were determined employing a calibration curve of bovine serum albumin (BSA) concentrations as the basis of the comparison. Before measurement, cells were lysed with 1 mol/L sodium hydroxide solution (15 min at room temperature) then 20 μL lysate was added to 200 μL Bradford reagent. The absorbance of the samples was measured at 595 nm using a plate reader.

### 3.8. Statistics

Means ± SEM values are derived from at least three independent measurements. Data were analyzed with Student’s t-test and one-way ANOVA using IBM SPSS Statistics software (version 21, Armonk, NY, USA) with *p* < 0.05 or *p* < 0.01 as the level of significance.

## 4. Conclusions

In summary, the interaction of resazurin and its reduced metabolite resorufin was investigated with native and chemically modified β-cyclodextrins, using fluorescence and UV-Vis spectroscopic, and molecular modeling studies. Each tested CD (BCD, HPBCD, and DIMEB) formed stable complexes with resazurin and resorufin, and the presence CDs led to the fluorescence enhancement of resorufin. These observations give rise to the conclusion that CDs can interfere with resazurin-based cell viability assay. In the present work, the testing of this hypothesis is described, using 30 or 120 min incubation times and cells undergoing co-treatment or separate treatment with resazurin and CDs. Furthermore, the effect of CDs on cell viability was examined also by quantifying intracellular ATP and total protein levels. The results presented suggest that CDs can indeed interfere with the resazurin-based cell viability assay, because CDs are able to affect the cellular uptake of resazurin. Furthermore, DIMEB (likely because of its cellular uptake) can enhance the fluorescence signal of resorufin, despite that the presence of DIMEB at increasing concentrations evokes the loss of viability of HepG2 cells. This study highlights that some CDs are able to return false results in the resazurin assay even if the cell medium is replaced prior to the cell viability assay. Therefore, the application of both CDs and the resazurin-based assay during cell experiments needs to be considered thoroughly.

## Figures and Tables

**Table 1:** Fluorescence enhancement (I/I_0_) of resorufin (0.4 μM) in the presence of cyclodextrins (CDs) (1200 μM each) at different emission wavelengths (λ_ex_ = 570 nm), and decimal logarithmic values of the binding constants (*K*; with the unit of L/mol) of resorufin-CD complexes at 25 and at 37 °C calculated from the fluorescent spectroscopic studies (λ_ex_ = 570 nm, λ_em_ = 583 nm; mean ± SD in 3 separate determinations).

	I/I_0_ (580 nm)	I/I_0_ (585 nm)	I/I_0_ (590 nm)	log*K* (25 °C)	log*K* (37 °C)
BCD	1.22 (±0.02)	1.30 (±0.02)	1.38 (±0.02)	3.31 (±0.08)	3.09 (±0.03)
HPBCD	1.01 (±0.01)	1.20 (±0.01)	1.39 (±0.02)	3.37 (±0.05)	3.26 (±0.05)
DIMEB	1.37 (± 0.04)	1.52 (±0.04)	1.53 (±0.03)	3.59 (±0.04)	3.52 (±0.05)

**Table 2:** Thermodynamic parameters of resorufin-BCD and resorufin-DIMEB complexes, ignoring the dehydration of guest molecules prior to the interaction (top), considering the outer dehydration of the guest molecules prior to the interaction (middle), or considering both the dehydration of the guest molecules and dehydration of the host’s cavity prior to the interaction (bottom).

Without dehydration prior to the interaction			
	Δ*H* (kJ/mol)	Δ*S* (J/K·mol)	Δ*G*_298 K_ (kJ/mol)
**resorufin-BCD**	−39.3	3.0	−40.2
**resorufin-DIMEB**	−50.5	1.9	−51.1
With dehydration of the guest prior to the interaction			
	Δ*H* (kJ/mol)	Δ*S* (J/K·mol)	Δ*G*_298 K_ (kJ/mol)
**resorufin-BCD**	−28.4	34.6	−38.8
**resorufin-DIMEB**	−39.6	33.5	−49.6
With dehydration of the guest and the host’s cavity prior to the interaction			
	Δ*H* (kJ/mol)	Δ*S* (J/K·mol)	Δ*G*_298 K_ (kJ/mol)
**resorufin-BCD ^1^**	−9.6	35.2	−20.9
**resorufin-DIMEB ^2^**	−13.2	34.1	−23.4

^1^ Theoretical log*K* value at room temperature was 3.52 while the experimental value was 3.33. ^2^ Theoretical log*K* value at room temperature was 4.09 while the experimental value was 3.55.

**Table 3:** Decimal logarithmic values of binding constants (*K*) of resazurin-CD and resorufin-CD complexes calculated from the changes of absorbance of the dye molecules (λ_resazurin-BCD_ = 605 nm, λ_resazurin-HPBCD_ = 607 nm, λ_resazurin-DIMEB_ = 611 nm, λ_resorufin-BCD_ = 575 nm, λ_resorufin-HPBCD_ = 577 nm, λ_resorufin-DIMEB_ = 579 nm; mean ± SD in 3 separate determinations).

	Resazurin-CD		Resorufin-CD	
	log*K*	λ_max_ (nm)	log*K*	λ_max_ (nm)
BCD	3.13 (± 0.02)	605	3.33 (± 0.02)	575
HPBCD	3.15 (± 0.04)	607	3.36 (± 0.03)	577
DIMEB	3.25 (± 0.03)	611	3.55 (± 0.05)	579

## Supplementary Materials

The following are available online at www.mdpi.com/link, Figure S1: Fluorescence excitation spectrum of resorufin (0.4 μM) in PBS (pH 7.4) [λ_em_ = 583 nm].
