# Supplementary material for: Complex Formation of Resorufin and Resazurin with Β-Cyclodextrins: Can Cyclodextrins Interfere with a Resazurin Cell Viability Assay?
Source: Molecules. 2018 Feb 10;23(2):382. doi: 10.3390/molecules23020382 (PMC6017802; doi:10.3390/molecules23020382)
Supplement: Supplementary file 1 [file molecules-23-00382-s001.pdf]

Complex Formation of Resorufin and Resazurin with Β-Cyclodextrins: Can Cyclodextrins Interfere with a Resazurin Cell Viability Assay?

SUPPLEMENTARY MATERIAL

Rita Csepregi [1,2], Beáta Lemli [2,3,4], Sándor Kunsági-Máté [2,3,4], Lajos Szente [5], Tamás Kőszegi [1,2], Balázs Németi [6], Miklós Poór [2,6*]

[1] Department of Laboratory Medicine, University of Pécs, Medical School, Pécs, H-7624, Hungary; ritacsepregi93@gmail.com (R.C.); koszegi.tamas@pte.hu (T.K.)
[2] János Szentágothai Research Center, University of Pécs, Pécs, H-7624, Hungary; lemli.beata@gytk.pte.hu (B.L.); kunsagi-mate.sandor@gytk.pte.hu (S.K.)
[3] Department of General and Physical Chemistry, University of Pécs, Pécs, H-7624, Hungary
[4] Department of Pharmaceutical Chemistry, University of Pécs, Faculty of Pharmacy, Pécs, H-7624, Hungary
[5] CycloLab Cyclodextrin Research & Development Laboratory, Ltd., Budapest, H-1097, Hungary; szente@cyclolab.hu
[6] Department of Pharmacology, University of Pécs, Faculty of Pharmacy, Pécs, H-7624, Hungary; balazs.nemeti@aok.pte.hu
* Correspondence: poor.miklos@pte.hu; Tel.: +36-72-536-000 Ext: 31646

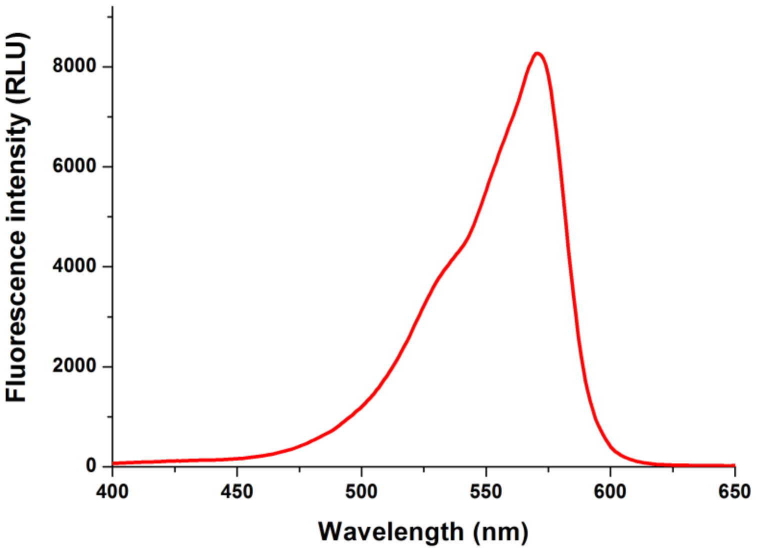

Figure S1. Fluorescence excitation spectrum of resorufin (0.4 μM) in PBS (pH 7.4) [λ_{em} = 583 nm].
